# Modeling and Simulation of a Machining Process Chain for the Precision Manufacture of Polar Microstructure

**DOI:** 10.3390/mi8120345

**Published:** 2017-11-27

**Authors:** Chenyang Zhao, Chi Fai Cheung, Mingyu Liu

**Affiliations:** Partner State Key Laboratory in Ultra-Precision Machining Technology, Department of Industrial and Systems Engineering, The Hong Kong Polytechnic University, Hung Hom, Kowloon, Hong Kong, China; Benny.Cheung@polyu.edu.hk (C.F.C.); samuel.liu@connect.polyu.hk (M.L.)

**Keywords:** ultra-precision machining, polar microstructure, process chain

## Abstract

This paper presents a functional microstructured surface, named Polar Microstructure. Polar microstructure is a three dimensional (3D) structured surface possessing a pattern of distribution of latitude and longitude micro-topographies with geometrical characteristics, which is similar to that in the Earth’s north or south pole. The spacing of its small surface features can achieve form accuracy at the micrometer level. Polar microstructure has great potential for applications in precision measurement of angle displacement based on the characteristics of its surface features. This paper presents the development of a machining process chain system that integrates single point diamond turning (SPDT) and diamond broaching together to fabricate polar microstructure. A framework of a machining process chain system is presented which is composed of input module, design module, simulation module, output module, and metrology module. After that, modeling of the machining process chain composed of SPDT and diamond broaching is built up. The model takes into consideration the initial surface topography of the workpiece. Simulations have been conducted to obtain the optimal machining parameters in each machining process. A series of experiments was conducted for the ultra-precision machining of various types of polar microstructures. The machining results show that the machining process chain system is technically feasible and effective in the precision manufacturing of polar microstructure. The experimental results agree well with the simulated results.

## 1. Introduction

Polar coordinates have been widely used in many fields, such as modeling, positioning, navigation, etc. [1]. For example, the Earth’s latitude and longitude system forms a polar coordinate scheme which is convenient for accurate positioning, and it is also easy to describe locations accurately with polar coordinates. 

In the precision engineering field, polar coordinates are also vital as they provide a potential method for positioning in precision measurement. For example, the working principle of most angular displacement sensors is that the rotation of the mechanical part is transmitted to the axis of the angular displacement of the sensor, so as to drive the spoiler/core connected to it, which then changes the induced voltage/inductance in the coil, and output of the voltage/current signal proportional to the rotational angle. An angular sensor can be a simple potentiometer or a more advanced Hall-Effect device [2,3,4,5]. 

It is interesting to note that the current measurement method of angular displacement relies heavily on other sensors that are more accurate. If a high precision micro-level polar coordinate is fabricated and determines the angular measurement through the computing vision method, there is no need for other sensors and the components of this measurement system are fewer in number. Moreover, angular displacement is able to be directly detected. To realize this measurement idea, the first step is fabrication of a high precision polar coordinate device.

This paper presents how to fabricate a high-precision polar coordinate device, known as polar microstructure, with the process chain machining system. In this paper, firstly the process chain system in ultra-precision machining is presented, which is composed of input module, design model, simulation module, output module, and metrology module. After that, some designs of polar microstructure are compared by using the flow of a machining process chain system, and an optimal machining process chain that combines single point diamond turning (SPDT) with diamond broaching is chosen after considering various factors. The next step is the modeling of the machining process chain. The initial surface topography of the workpiece is also modeled to make the accuracy of the whole process chain model higher. Simulations are undertaken under different machining parameters in each machining process and one group of machining parameters was chosen to conduct the verification machining experiment. After measuring the machining quality of the sample, it can be concluded that the simulation results agree well with the actual experimental results. 

## 2. Machining Process Chain System

**Figure 1:**
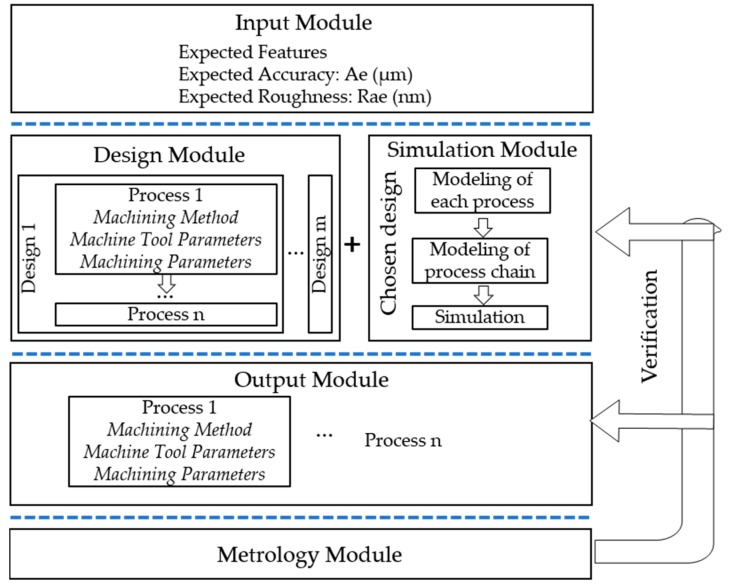
Framework of a machining process chain system.

The machining process chain is an emerging area in precision manufacturing. Thompson [6] applied it for the selection of an additive manufacturing context, and Uhlmann [7] presented a review of the process chain research regarding high-precision components with micro-scale features. The authors [8] proposed a process chain optimization system in ultra-precision machining, and then a process-chain based study [9] was conducted to investigate the influence of the machining parameters in ultra-precision raster milling. A framework of a machining process chain system is shown in Figure 1. 

In the input module, expected features of the workpiece should be entered. Moreover, the required accuracy and surface quality requirement are also needed to be given. Afterward, some initial machining process chains are presented in the design module, considering the limitation of each designated process chain, such as its form accuracy, surface roughness, machining efficiency, etc. An optimal machining process chain includes machining methods and relevant parameters that are selected. 

After that, in simulation module, the modeling of individual machining processes is conducted and the machining process chain is modeled based on the results of the previous modeling of the individual machining process. Simulation is undertaken so as to obtain the predicted performance of the selected process chain. The workpiece is then machined based on the parameters in the output module. In the metrology module, the processed workpiece is measured so as to verify whether the results meet the requirements. If they do not, a repeated cycle starting from the design module to metrology module should be conducted until all of the indicators are acceptable.

## 3. Design of Polar Microstructure

Based on the abovementioned process chain system in ultra-precision machining, there are different methods to machine polar microstructure. At the first stage, the objective of machining quality of polar microstructure should be discussed. Polar microstructure is designed in an attempt to be applied in encoding precision measurement, which aims to achieve nanometer-level resolution, its surface asperities should achieve nanometer level while the form accuracy should be micrometer-level form accuracy. When considering the above requirements, ultra-precision machining technology is indispensable to satisfy the stringent requirements. 

During the design module, three possible machining chains are shown, as seen in Table 1. In machining process chain 1 (MPC1), fast tool servo (FTS) machining suffers from complicated program coding. Moreover, the FTS is usually limited by the size of the workpiece, which should not be too large. Machining process chain 2 (MPC2) makes use of both SPDT and Ultra-Precision Raster Milling (UPRM), which overcomes the shortcomings of MPC1. However, it is difficult to align with two machining centers due to repositioning error. Moreover, the depth of cut is also difficult to be controlled well after repositioning. 

**Figure 2:**
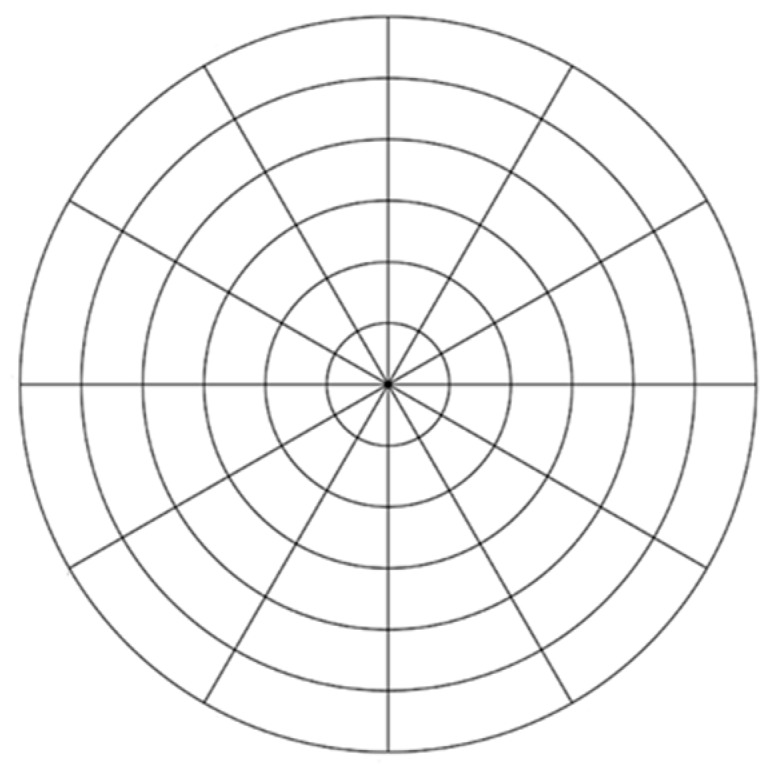
Design of machining process 3 (MPC3).

Comparatively, Machining process 3 (MPC3) combines SPDT with diamond broaching together, and its machining principle is shown in Figure 2. The concentric grooves are machined by SPDT and the straight grooves are machined by diamond broaching with the same machine tool (Moore Nanotech 350FG, Nanotechnology Inc, Swanzey, NH, USA) as that of SPDT. It overcomes the above shortcomings in MPC1 and MPC2. More importantly, MPC 3 has the ability to machine large-sized workpieces with high efficiency. Hence, the process chain of machining polar microstructure is determined. The next step is to determine the machining parameters in each machining process, modeling of the process chain and the corresponding simulation necessary to realize it.

## 4. Modeling of the Machining Process Chain

**Figure 3:**
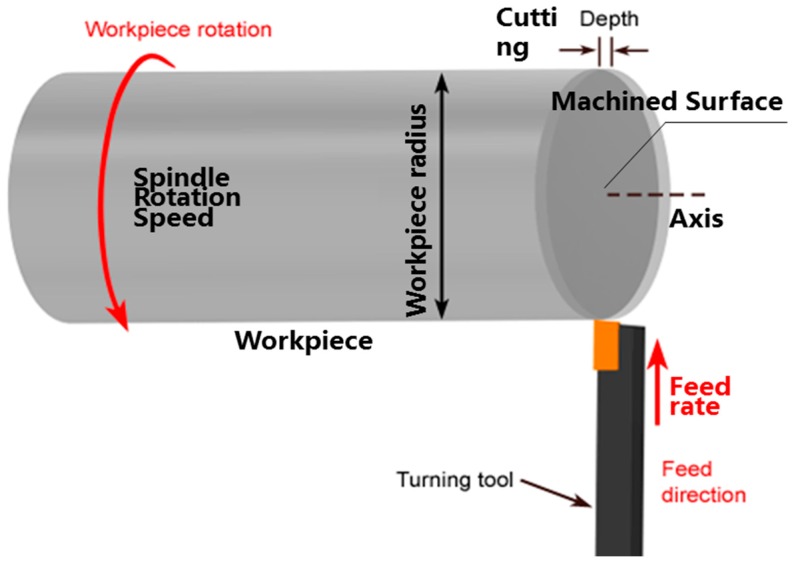
Processing principle of single point diamond turning (SPDT).

The initial workpiece surface before machining by MPC3 is pre-machined by SPDT; the first modeling step aims to build up the initial surface topography of the workpiece before MPC3. The SPDT process is shown in Figure 3. The workpiece is mounted and rotated with the spindle of the machine tool, the diamond cutting tool feeds along the radial direction of the workpiece with a certain depth of cut, and the materials on the surface are removed by a diamond tool at a high cutting speed. 

**Figure 4:**
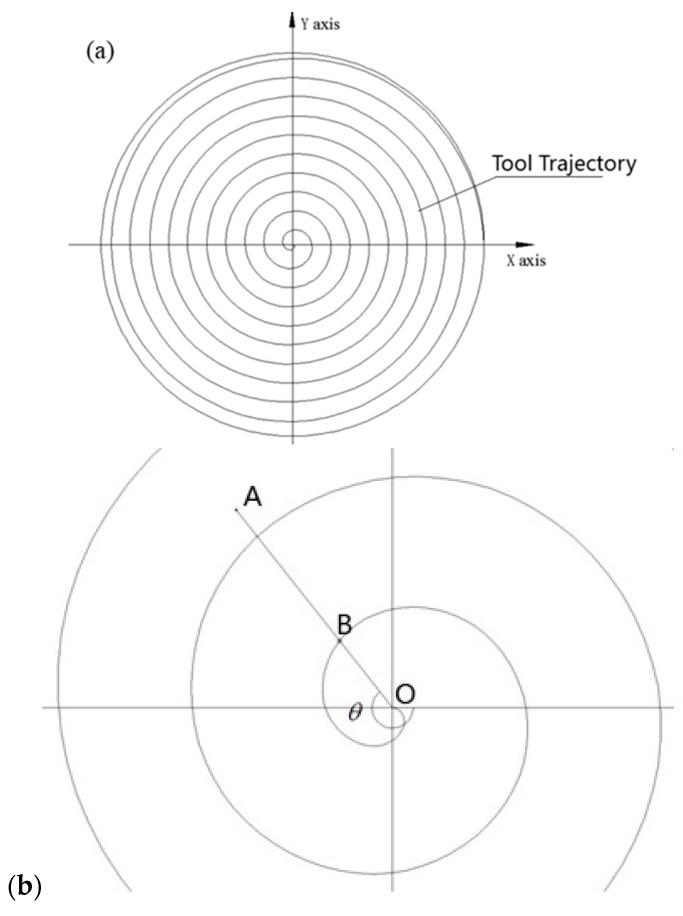
(**a**) Diamond tool trajectory of single point diamond turning (SPDT) on workpiece surface (**b**) Enlarged top view.

Figure 4a shows the tool generation trajectory of SPDT on the workpiece surface. Because of the rotation of the workpiece, as well as the feed of the diamond tool, a spiral line trajectory is shaped. As shown in Figure 4b, *O* is the center point of workpiece, *A* is an arbitrary point (x,y,z) on the machined surface, *B* is the intersection point of the line *OA* and the first-circle spiral line, and *θ* is the angle from the *X* positive axis to line *OB*. Based on the geometrical relationship, it can be derived that:

(1) $$\theta =\arctan(\frac{y}{x})$$

(2) $$t_{1}=\frac{\theta }{2\pi (\omega /60)}=\frac{30\theta }{\pi \omega }$$

(3) $$r_{OB}=\frac{f\cdot t_{1}}{60}$$

where *θ* is the angle from the *X* positive axis to line *OB*, $t_{1}$ is the time period that the tool nose moves from point *B* to point *O*, *f* is the feed rate of the diamond tool, *ω* is the spindle speed of the machine tool, and $r_{OB}$ is the distance between *O* and *B*.

**Figure 5:**
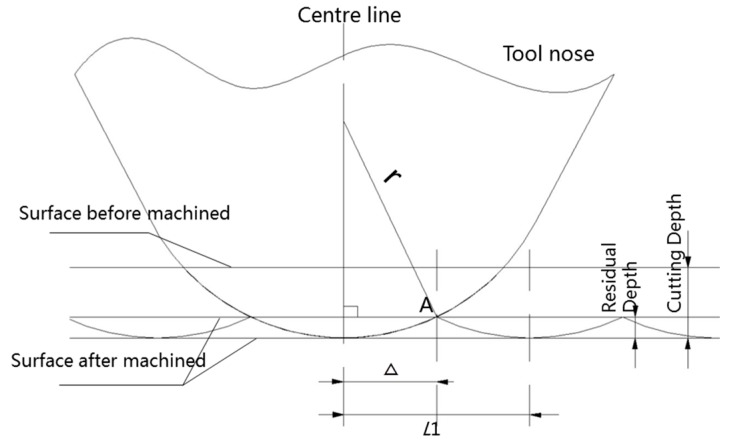
Geometrical model of the surface topography machined by SPDT.

The geometrical relationship between the tool nose and the workpiece is shown in Figure 5. The surface topography of the workpiece is mainly affected by the tool nose radius and machining parameters, such as spindle speed, feed rate, etc. As shown in Figure 5, *r* represents the tool nose radius. *L*_1_ is the feed distance per revolution, and it can be presented by the equation:

(4) $$L_{1}=\frac{f}{\omega }$$

*Δ* is the distance from point A(x,y,z) to the center line of the tool nose, which can be derived as:

(5) $$\Delta =|\sqrt{x^{2}+y^{2}}-(r_{OB}+K\cdot L_{1})|$$

where *K* is the length of the period from point A to point O.

According to the right triangle side length relationship, the height $z_{SPDT}$ of point A is derived:

(6) $$z_{SPDT}=(r-d_{1})-\sqrt{r^{2}-\Delta^{2}}$$

where $d_{1}$ is the depth of cut.

At this point, the surface topography model of the workpiece machined by SPDT has been built. The model is not only built up for machining concentric grooves, but also for the surface topography of pre-machined process machined by SPDT. In order to separate the parameters, $z_{pre}$ represents the surface height of the workpiece in the pre-machining process, and $z_{con}$ represents the surface height of the workpiece resulting from machining concentric grooves. As for the broaching process, the modeling method is similar to that of SPDT; it aims to obtain the surface height $z_{str}$ resulting from the broaching process.

For the modeling of the whole of the process chain, the final surface topography results from all of the previous machining processes, for the arbitrary point of workpiece, its surface height $z_{chain}$ depends on the lowest height of $z_{pre}$, $z_{con}$ and $z_{str}$, which can be defined as follows:

(7) $$z_{chain}=\arg\underset{(x,y)\in W}{\max}\{z_{pre},z_{con},z_{str}\}$$

where W is the whole area of the workpiece.

At this point, the model of the whole machining process chain has been built up.

**Figure 6:**
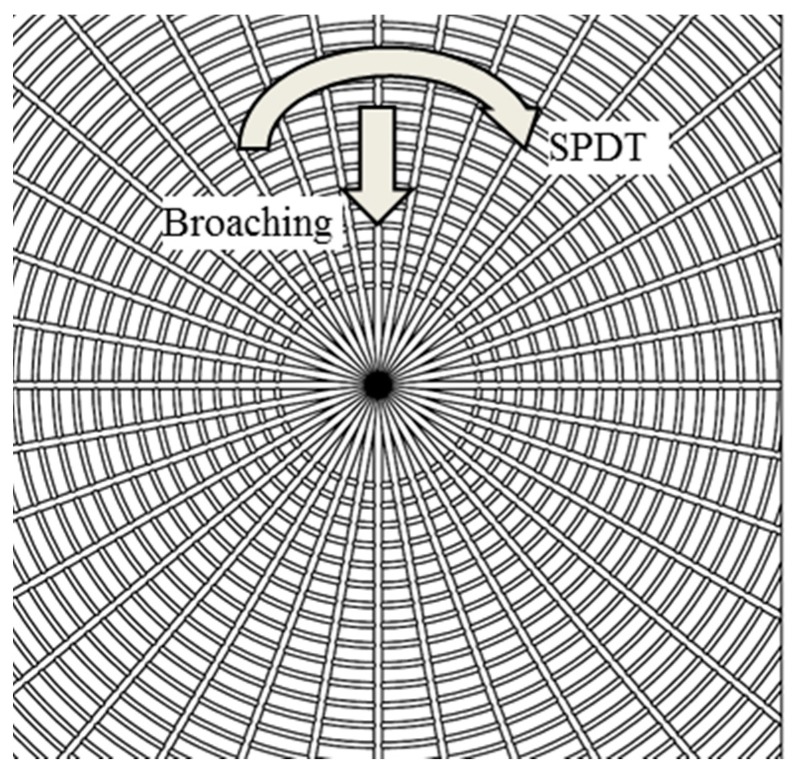
MPC3 (Combining SPDT and diamond broaching process) of machining polar microstructure.

**Figure 7:**
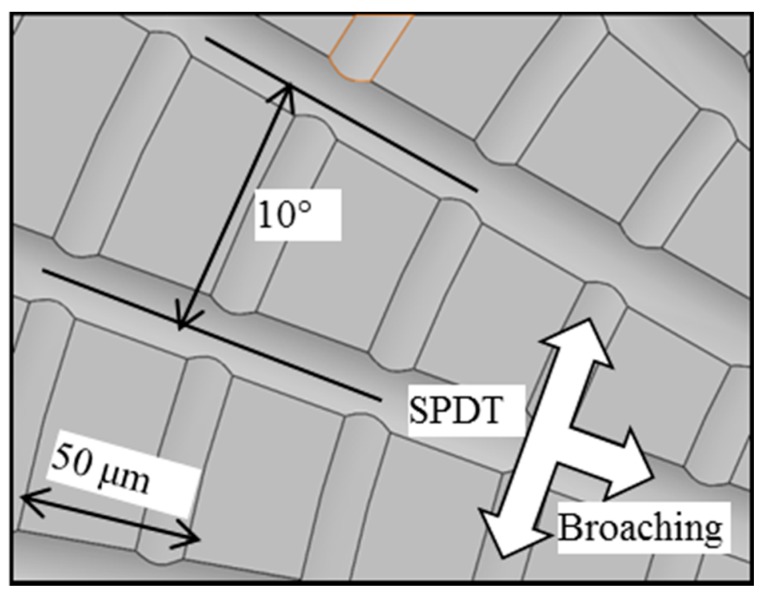
Enlarged top view of the process chain model for machining polar microstructure.

Figure 6 shows the simulation of MPC3. Unlike the previous SPDT machining method, the diamond tool has no feed speed along the radial axis of the workpiece in MPC3. Alternatively, the diamond tool feeds a step forward, and then the circle grooves are machined by SPDT. As for the straight grooves, they are machined by diamond broaching, which makes use of the same diamond tool and machine tool to machine the surface, which is able to eliminate repositioning errors when the machining process changes. The diamond tool broaches the workpiece surface with uniform angle stepping, the broaching center of workpiece coincides with that of SPDT, and the final modeling effect of polar microstructure is shown in Figure 7. The feature of polar microstructure is mainly determined by the machining parameters in MPC3, such as step distance, tool nose radius, etc.

Based on the models of SPDT and diamond broaching that have been built, a series of simulation experiments was conducted by using different machining parameters. A group of optimal parameters are shown in Table 2 and the simulated surface texture of the polar microstructure is shown in Figure 7. The distance and angle spacing of its texture are chosen to be 50 μm and 10°, respectively, after considering both machining tool accuracy and material properties of the workpiece.

## 5. Experimental and Simulated Results

A series of cutting experiments with the machining parameters based on the optimal designed ones by the process chain system was conducted. In the experiment, polar microstructure was machined by Nanoform 350G from Nanotechnology Inc., Swanzey, NH, USA. In the meanwhile, the corresponding models were also constructed and were used to simulate the machining process chain. 

**Figure 8:**
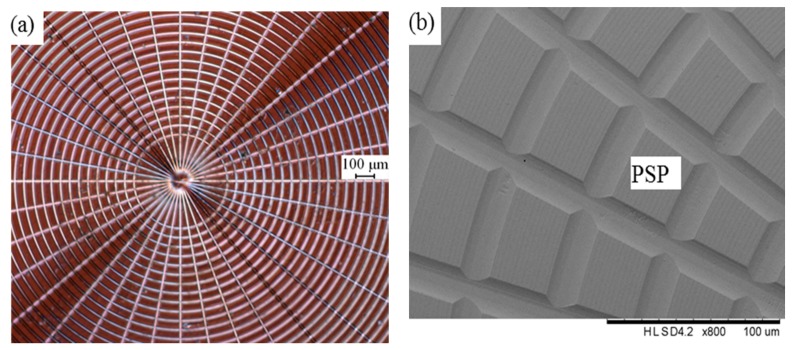
Polar microstructure measured by (**a**) Pearl Centering Microscope (**b**) Hitachi Electron Microscope TM3000.

The experimental results of the workpiece surface topography are shown in Figure 8. As shown in Figure 8a, the intersection of the straight grooves coincides well with the center of concentric grooves. There are 36 straight grooves that are evenly distributed on the surface of the polar microstructure.

Moreover, the distance between each adjacent concentric groove is 50 μm. To clearly observe polar microstructure, the workpiece was measured by a Scanning Electron Microscope (SEM) (Hitachi Electron Microscope TM3000, Hitachi, Tokyo, Japan), as shown in Figure 8b. The surface quality of the workpiece is good, including cutting grooves as well as the protruding surface parts (such as the ‘protruding surface parts (PSP)’ marked in Figure 8b), which were not machined in this process chain.

**Figure 9:**
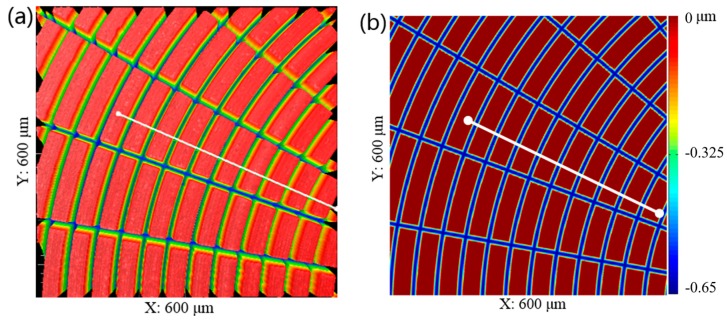
Surface topography of the workpiece machined by MPC3 (**a**) Measured with Zygo Nexview™ 3D Optical Surface Profiler (**b**) Simulated by Matlab 2017.

**Figure 10:**
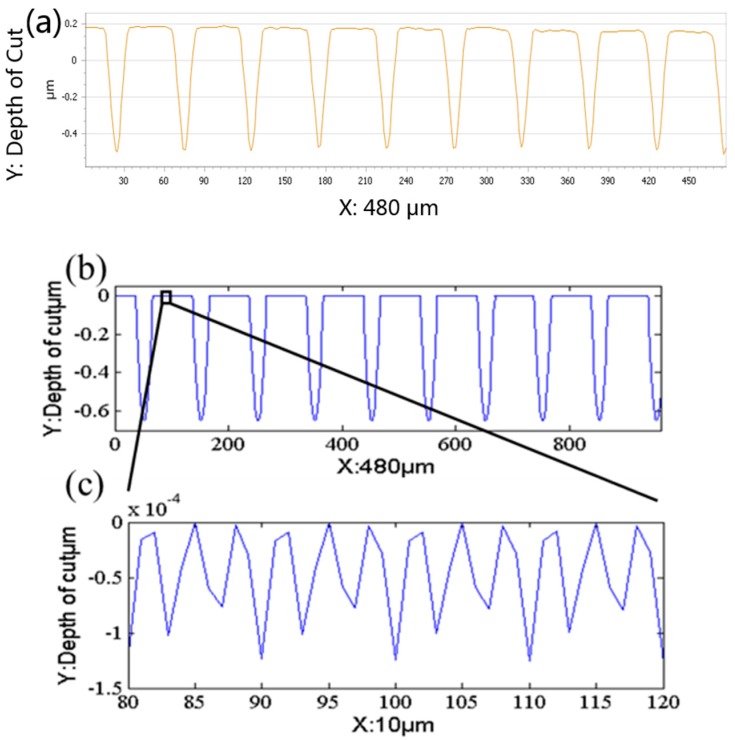
Comparison between the model and machining performance of the polar microstructure (**a**) Sectional view to show the structure space and depth of cut measured by Zygo, (**b**) The structure space and depth of cut simulated by surface topography model, (**c**) Enlarged sectional view of (**b**).

To illustrate the experimental results through the specific data, the workpiece was also measured with a Zygo Nexview™ 3D Optical Surface Profiler (Zygo, Middlefield, CT, USA), as shown in Figure 9a, and the corresponding simulation is shown in Figure 9b. A comparison of structure spacing and depth of cut between model and machining performance of the polar microstructure is shown in Figure 10.

To study the factors affecting the accuracy of the polar microstructure, a total of 50 protruding surface parts (PSP) were included in this measurement experiment. Some parameters of the factors was recorded. It should be illustrated that not only the arithmetic roughness (*R_a_*) on the PSP were recorded, but also 50 straight grooves and 50 round grooves between neighboring PSPs were recorded. The factors under investigation included (1) the cutting depth of machined grooves; (2) the spacing between adjacent grooves; and, (3) the surface roughness (*R_a_*) of PSP (protruding surface parts) marked in. The experimental results are shown in Table 3. When compared with the designed machining parameters, as shown in Table 2, SG_A_ is 0.02 μm less than the designed spacing distance, DCS_A_ and DCR_A_ are 22.3 nm and 34.6 nm, respectively, more than the designed depth of cut, and DCS_S_ and DCR_S_ are 20.5 nm and 28.9 nm, respectively. Moreover, *R_a_* of PSP is 14.6 nm, which infers a high surface quality of the polar microstructure.

**Figure 11:**
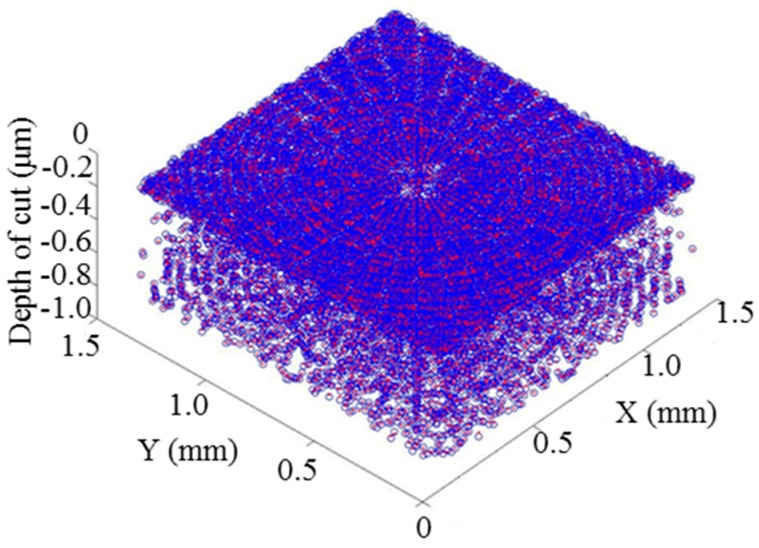
Z axis registration result. The blue color-coded surface represents the designed surface and the red colour-coded surface represents the experimental surface.

**Figure 12:**
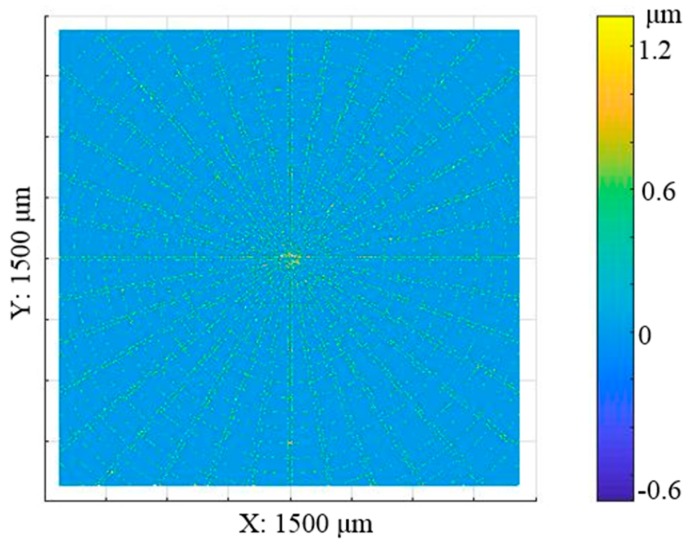
Error map compared with the design surface.

The next step was to register the datasets in the z direction. The process aims to search for the minimum distance between the simulated surface and experimental machined surface by using an iterative closest point (ICP) method. The final stitching result is shown in Figure 11. The result shows that the two surfaces are well registered in the z direction. Moreover, the error map is determined, as shown in Figure 12.

The root-mean-square (RMS) value of the error map is 0.078 μm. The error map shows that the error is evenly distributed and in most of the areas, it is nearly zero. It is also interesting to note that the error in the central area is relatively larger as compared with that in the surrounding areas. This is mainly caused by the accumulated machining errors of the broaching process. Besides, larger errors are mainly distributed corresponding to the machining tool path, which may be due to the plowing phenomenon of materials when the diamond tool is machining the surface. When compared to the SPDT process, the machining error in the area machined by the diamond broaching process is more obvious. This is partly due to the relatively low broaching speed as compared with the cutting speed of SPDT, but with the constant spindle speed, the cutting speed of SPDT in the area to the center is lower and lower; if the error map results are exactly explained by the above, the closer to the workpiece center, the larger that the error caused by SPDT should be. However, Figure 12 shows that errors are distributed evenly. Another reason why the machining error in the area machined by the diamond broaching process is more obvious, and this is probably because of the differences in the depth of cut. According to the measured depth of cut values, as shown in Table 3, there is a difference of 12.3 nm in terms of the arithmetic roughness value between SPDT and broaching, which may influence the material removal process and thereby the error map as compared with the design surface. As a result, further research should be conducted to investigate this. 

## 6. Conclusions

This paper presents a machining method for the precision manufacturing of polar microstructure based on a machining process chain system in ultra-precision machining. Polar microstructure has potential applications for precision measurement, while the machining accuracy and surface quality are also key factors that need to be considered. 

In this paper, a framework of machining process chain system is presented and the design flow was described. Based on the process chain system, the optimal process chain for machining polar microstructure was selected, followed by the determining of machining parameters. 

A series of experiments was conducted, according to the designed machining parameters followed by simulations. The experimental results show that the accuracy of different machining parameters that influence the future performance of polar microstructure is in the nanometer range. 

The result shows that a machining process chain method, which combines SPDT with diamond broaching, is technically feasible and efficient for the precision manufacture of polar microstructure. The results also infer that the machining process chain system plays an important role for designing and optimizing the machining processes in ultra-precision machining.

## Figures and Tables

**Table 1:** Different machining process chains for manufacturing polar microstructure.

Process Chain	Machining Processes
Process Chain 1	Fast Tool Servo (FTS) Machining
Process Chain 2	SPDT + Ultra-precision Raster Machining (UPRM)
Process Chain 3	SPDT + Diamond Broaching

**Table 2:** Machining parameters in the manufacturing of micro polar structure.

Machining Parameters	SPDT in the Initial Machining Process	SPDT in MPC3	Broaching in MPC3
Workpiece material	Nickel-Copper		
Radius of the diamond tool	2.504 mm	0.043 mm	0.043 mm
Spindle speed of SPDT	6000 r/min	2000 r/min	N/A
Feed rate of SPDT	5 mm/min	N/A	N/A
Feed rate of broaching	N/A	N/A	600 mm/min
Depth of cut	2 μm	0.65 μm	0.65 μm
Spacing	N/A	50 μm	50 μm

N/A: Not applicable.

**Table 3:** Experimental parameters influencing the machining quality of the polar microstructure and relevant simulated results.

Indicators of machining quality	Experiment	Simulation
Average depth of cut in straight grooves (DCSA)	672.3 nm	650 nm
Standard deviation of depth of cut in straight grooves (DCSS)	20.5 nm	N/A
Average depth of cut in round grooves (DCRA)	684.6 nm	650 nm
Standard deviation of depth of cut in round grooves (DCRS)	28.9 nm	N/A
Average spacing between adjacent grooves (SGA)	49.98 μm	50 μm
Arithmetic Roughness (*R_a_*) of protruding surface parts (PSP)	14.6 nm	12 nm

N/A: Not applicable.

## References

[1] Aidala V., Hammel S. (1983). Utilization of modified polar coordinates for bearings-only tracking. IEEE Trans. Autom. Control.

[2] Van Schoiack M.M., Mawet P.H. (1988). Angular Displacement Sensor. U.S. Patent.

[3] Silas K. (1967). Angular Displacement Sensor. U.S. Patent.

[4] Sikkens B.T. (2007). Angular Displacement Sensor. U.S. Patent Application.

[5] Gentile C.T., Wallace M., Avalon T.D., Goodman S., Fuller R., Hall T. (1992). Angular Displacement Sensors. U.S. Patent.

[6] Thompson M.K., Stolfi A., Mischkot M. (2016). Process chain modeling and selection in an additive manufacturing context. CIRP J. Manuf. Sci. Technol..

[7] Uhlmann E., Mullany B., Biermann D., Rajurkar K., Hausotte T., Brinksmeier E. (2016). Process chains for high-precision components with micro-scale features. CIRP Ann. Manuf. Technol..

[8] Zhao C., Cheung C.F., Kong L. A study of optimization of machining strategy for enhancing the efficiency of process chain in ultra-precision machining. Proceedings of the Euspen’s 16th International Conference & Exhibition.

[9] Zhao C., Cheung C.F., Liu M. A study of influence of machining parameters on process chain in ultra-precision raster milling. Proceedings of the 5th International Conference on Nano Manufacturing (nanoMan 2016).

